# Granulocyte colony-stimulating factor effects on neurological and motor function in animals with spinal cord injury: a systematic review and meta-analysis

**DOI:** 10.3389/fnins.2023.1168764

**Published:** 2023-06-28

**Authors:** Jing-Wei Tao, Xiao Fan, Jing-Ya Zhou, Lu-Yao Huo, Yan-Jun Mo, Hui-Zhong Bai, Yi Zhao, Jing-Pei Ren, Xiao-Hong Mu, Lin Xu

**Affiliations:** ^1^Center for Orthopedic Surgery, Beijing University of Chinese Medicine Dongzhimen Hospital, Beijing, China; ^2^Beijing University of Chinese Medicine, Beijing, China; ^3^Qingdao Municipal Hospital, University of Health and Rehabilitation Sciences, Qingdao, China

**Keywords:** spinal cord injury, neurological function, Basso, Beattie, and Bresnahan scale score, granulocyte colony-stimulating factor, optimal dosage

## Abstract

**Background:**

Spinal cord injury (SCI) is a severe neurological injury for which no effective treatment exists. Granulocyte colony-stimulating factor (G-CSF) is used to treat autologous bone marrow transplantation, chemotherapy-induced granulocytopenia, Acquired Immune Deficiency Syndrome (AIDS), etc. Recent research has revealed the potential application of G-CSF on neuroprotective effectiveness. In central nervous system diseases, G-CSF can be used to alleviate neuronal injury.

**Objective:**

To investigate the effects of G-CSF on Basso, Beattie, and Bresnahan (BBB) scale score, inclined plane test, electrophysiologic exam, quantitative analysis of TUNEL-positive cells, and quantitative analysis of glial fibrillary acidic protein (GFAP) immunostaining images in animal models of SCI.

**Methods:**

We searched PubMed, Web of Science, and Embase databases for all articles on G-CSF intervention with animal models of SCI reported before November 2022. A total of 20 studies met the inclusion criteria.

**Results:**

Results revealed that G-CSF intervention could improve the BBB scale score in both groups at 3, 7, 14, 28, and 35 days [at 35  days, weighted mean differences (WMD) = 2.4, 95% CI: 1.92–2.87, *p* < 0.00001, I^2^ = 69%]; inclined plane test score; electrophysiologic exam; quantitative analysis of TUNEL-positive cell numbers; quantitative analysis of GFAP immunostaining images in animal models of SCI. Subgroup analysis revealed that treatment with normal saline, phosphate-buffered saline, and no treatment resulted in significantly different neurological function effectiveness compared to the G-CSF therapy. SD rats and Wistar rats with SCI resulted in significant neurological function effectiveness. C57BL/6 mice showed no difference in the final effect. The T9–T10 or T10 segment injury model and the T8–T9 or T9 segment injury model resulted in significant neurological function effectiveness. The BBB score data showed no clear funnel plot asymmetry. We found no bias in the analysis result (Egger’s test, *p* = 0.42). In our network meta-analysis, the SUCRA ranking showed that 15 mg/kg-20 mg/kg was an optimal dose for long-term efficacy.

**Conclusion:**

Our meta-analysis suggests that G-CSF therapy may enhance the recovery of motor activity and have a specific neuroprotective effect in SCI animal models.

**Systematic review registration**: PROSPERO, identifier: CRD42023388315.

## Introduction

A spinal cord injury is often caused by severe trauma. It causes significant negative impairments in the patient’s quality of life (Eckert and Martin, 2017). It results in the patient’s loss of motor function and induces multisystem complications. SCI usually progresses through two stages of pathology. The first stage is spinal cord contusion trauma. It typically consists of the rupture of blood vessels and cell membranes. The second stage is the pathophysiological response and typically involves ischemia, apoptosis, and hypoxia (Zhang et al., 2022). SCI results in massive social, physical, and lifelong healthcare costs to the community and society (Chen et al., 2022). The incidence of SCI ranges from approximately 10.4 parts per million to 83 parts per million persons annually worldwide (Eli et al., 2021). SCI is highly likely to result in disability. In the United States, 40 to 50% of cervical spine injuries result in complete tetraplegia (Hall et al., 2019). The extent of disability for patients depends on the type of injury. It was anticipated that a person with SCI would incur $1,130,000 in direct costs in the first year after the accident, followed by $196,107 annually thereafter (Lo et al., 2021). According to the American Association of Neurological Surgeons (AANS) and Congress of Neurological Surgeons (CNS) joint section guideline series and an AOSpine guideline (Walters et al., 2013). Patients with SCI should be transferred to a specialized medical center for treatment as soon as possible. Maintain basic vital signs and avoid systemic hypotension caused by hypovolemia and sympathetic nerve fiber injury. Transport with a cervical collar or backboard to prevent further injury to the spinal cord. The lack of effective treatments for SCI imposes a significant economic and healthcare system burden (Huang et al., 2020). With the development of spinal instrumentation, surgical decompression can effectively improve nerve damage from hemorrhage (Ahuja et al., 2017). Nevertheless, most patients have difficulty accessing appropriate pre-hospital assessment and surgical timing (Rouanet et al., 2017). Therefore, clinical treatment with neuroprotective drugs is mostly used. Some steroids, most notably methylprednisolone, have been reported to reduce secondary injuries. However, some clinical trials have reported negative results (Shank et al., 2019). The incidence of adverse events increases when methylprednisolone is administered in excessive amounts (Liu et al., 2019). To treat spinal cord injuries, it is therefore required to identify pharmacological candidates with pleiotropic characteristics and multiple action mechanisms.

The G-CSF gene is 2.5 kb in size, and it includes five exons and four introns. G-CSF could improve the proliferation, differentiation, and activation of neutrophils. The Food and Drug Administration approves G-CSF, and its indications are autologous bone marrow transplantation, chemotherapy-induced granulocytopenia, AIDS, etc (Wallner et al., 2015) According to their mechanism of action, G-CSF is divided into granulocyte-derived and granulocyte-macrophage-derived forms. Recombinant human granulocyte colony-stimulating factor (rhG-CSF) acts on hematopoietic progenitor cells to promote their proliferation and differentiation (Inada et al., 2014). In recent studies, G-CSF has been demonstrated to have multiple therapeutic potentials for central nervous system (CNS) injury. Several researchers have investigated the role of G-CSF receptors in neuroprotective function in the CNS (Wallner et al., 2015). Numerous studies in SCI animal models have shown that G-CSF restores microglial function, reduces apoptosis, attenuates neuroinflammation, and promotes motor function recovery (Aschauer-Wallner et al., 2021). The pharmacological profile of G-CSF is well characterized, its mode of action is pleiotropic, and it has the potential to be used over a long period of time. While some researchers have observed that G-CSF does not influence motor function, they have found that it promotes the recovery of tactile abilities (Thomaty et al., 2017).

Researchers have conducted several studies to confirm the clinical effectiveness of G-CSF on neurological recovery in patients with traumatic SCI (Derakhshanrad et al., 2013; Saberi et al., 2014; Kamiya et al., 2015). Nevertheless, high-quality research remains rare. A meta-analysis based on randomized controlled trials cannot be completed. Currently, there is no published meta-analysis on G-CSF as a treatment for SCI. Therefore, providing preclinical evidence of the effectiveness of this treatment for subsequent human clinical trials is necessary.

### Literature search

Our meta-analysis was conducted according to PRISMA guidelines (Page et al., 2021). Two investigators (TJW and FX) independently searched the Web of Science, PubMed, Embase databases, and the Cochrane Library for all studies investigating G-CSF intervention in SCI animals reported before November 2022. The search strategy was as follows: [spinal cord injury OR spinal cord injuries OR spinal cord trauma OR spinal cord transection OR spinal cord laceration OR spinal cord contusions OR post-traumatic myelopathy] AND [G-CSF OR granulocyte colony-stimulating factor] AND [animals]. The list of citations included in the literature was manually searched to obtain additional data.

### Selection criteria

We screened the studies that met the inclusion criteria: (1) separate G-CSF treatment in research using an SCI animal model, (2) studies in laboratory animals with SCI of any age, sex, species, or strain, (3) studies that induced SCI in animal models by methods such as compression, hemisection, transection, or contusion, etc., (4) studies that used G-CSF intervention with any delivery technique, dose, or composition of G-CSF-derived substance, (5) studies with control methods that included physiological saline, excipient, phosphate-buffered saline (PBS), or no treatment, and (6) studies that recorded motor function score, GFAP levels, and apoptotic cell number as outcomes. Studies that met the following exclusion criteria were removed: (1) *in vitro* research or clinical studies, (2) systematic reviews, meta-analyses, and conference reports, (3) uncontrolled experimental studies, (4) full-text studies not available, (5) studies with additional intervention without G-CSF, (6) *in vitro* studies, clinical studies, and studies without a control group or a separate granulocyte colony-stimulating factor group, and (7) studies without data on behavioral scores, GFAP levels, and apoptotic cell counts.

### Data extraction

Two members (ZJY and BHZ) individually extracted pertinent data from the included studies: authors, publication year, animal species and sex, SCI model, traumatic level, intervention, dose, administration time, administration route, administration frequency, and control method. The other authors (HLY and MYJ) resolved any disagreement between the first two authors.

### Risk of bias and assessment of the methodological quality

Two reviewers (ZY and RJP) individually evaluated the risk of bias and methodological quality. The Systematic Review Centre for Laboratory Animal Experimentation’s Risk of Bias tool (SYRCLE’s RoB tool) (Hooijmans et al., 2014) was utilized to assess the methodological quality of the included animal studies. The list of SYRCLE’s RoB tool consists of 1. selection bias, 2. performance bias, 3. detection bias, 4. attrition bias, 5. reporting bias, and 6. other bias. The risk was assessed as a “yes” or “no” judgment signal, and “unclear” means that details were insufficient to evaluate the risk.

### Outcome measures

Motor function was assessed and recorded by pooling data from the BBB scores, motion-evoked potential, somatosensory-evoked potential, and inclined plane scores. Neurological function was assessed by GFAP levels and apoptotic cell counts. BBB scale data were used as the primary outcome. Inclined plane scores, motion-evoked potential, somatosensory evoked potential, GFAP levels, and apoptotic cell counts were considered secondary outcomes.

The BBB Locomotor Rating Scale is a behavioral score used to measure a rat’s hindlimb motor function. By observing plantar stepping patterns and joint movements, the BBB score indicates the severity of the injury. A score of 0–21 is used to indicate the degree of recovery from SCI, with 0 points classified as having no hindlimb motor function and 21 points classified as being able to move normally (Basso et al., 1995).

The inclined plane test is a test that assesses motor function by observing the maximum angle at which a rat can stay on an inclined plane. The greater the angle at which the rat remains on the inclined plane for more than 5 s, the better the recovery of motor function after SCI (Zhou et al., 2022).

A hindlimb electrophysiological test is a method of evaluating hindlimb muscle function by inserting electrodes into the somatosensory and motor cortex areas of SCI rats.

TUNEL-positive cell counts of spinal cord tissue as apoptotic cell numbers. Quantitative analysis of GFAP immunofluorescence intensity was defined as GFAP level.

### Statistical analysis

The meta-analysis was completed and combined using Review Manager (version 5.3). GraphPad Prism (version 9) software was used to draw graphs. WMD and 95% confidence intervals (CIs) were used for outcomes measured with the same unit. The *I*^2^ index was utilized to perform a homogeneity analysis. Results with high heterogeneity were analyzed with random-effects models (*I*^2^ ≥ 50%), and results with low heterogeneity were analyzed with fixed-effect models (*I*^2^<50%). Publication bias was assessed by a funnel plot, and asymmetry was confirmed using Egger’s regression. Furthermore, we performed multiple subgroup studies to identify the origins of heterogeneity, such as control group treatment method, species, SCI segment, and administration dose. Stata (version 14.0) was used to perform the network analysis comparing direct or indirect treatment regimens to determine the optimal dosage of G-CSF. Effective doses were then ranked according to SUCRA values.

## Results

### Search results and study characteristics

**Figure 1:**
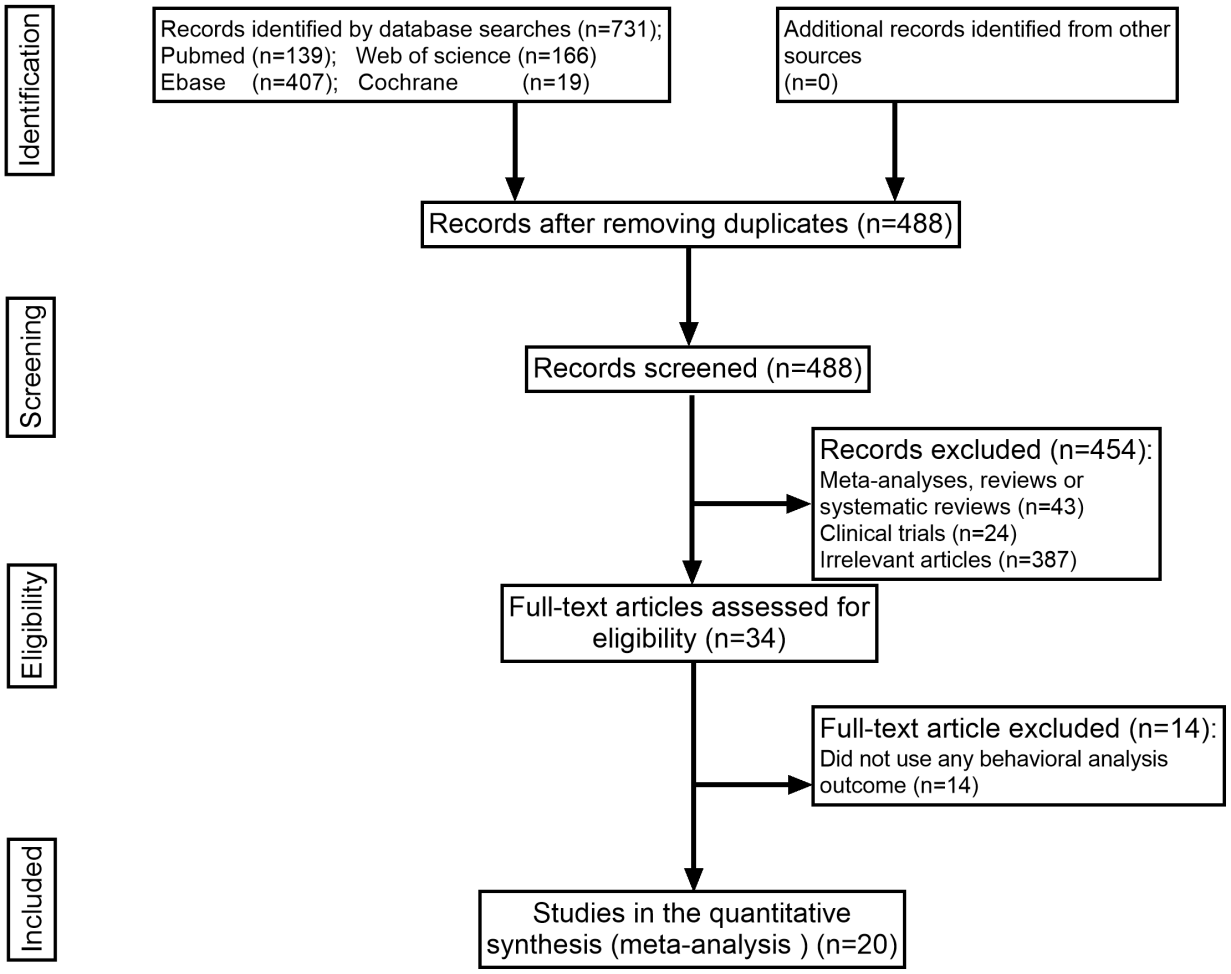
Flow chart of the literature selection process.

The search strategy identified 731 items in total, 243 of which were duplicates. After title and abstract screening, 34 articles were retained. Of the 14 articles excluded from the full-text review, 20 that met the inclusion requirements were retained. Of the 14 articles excluded from the full-text review, all of them did not apply any behavioral analysis outcome. All publications that met the predetermined inclusion criteria were searched in the English-language database (Figure 1).

In the included literature, the minimum sample size was 20 animals, and the maximum sample size was 120 animals. A total of 11 studies used Sprague–Dawley rats, four studies used Wistar rats, two studies used Kunming mice, two studies used C57BL/6 mice, and only one article used BALB/c mice. A total of 10 studies used the spinal cord contusion model, and four studies used the spinal cord hemisection model. Four studies reported on the vascular clip-induced compression damage model. A balloon compression injury was employed as a model in one investigation. Only one study induced a contusive SCI by applying a base plate over the dura mater. Two articles indicated injury at the T8 or T7–T8 level; 10 studies reported injury at the T9 or T8–T9 level; and eight publications reported trauma at the T10 or T9–T10 level. Animals in three studies received treatment with recombinant human GM-CSF, and those in 17 studies received treatment with G-CSF. Control groups received the vehicle, phosphate-buffered saline (PBS), physiological saline, or no treatment (Table 1).

**Table 1:** Characteristics of all included studies.

Study	Strain	Sex	Model	Segment	Colony Stimulating Factor Source	Dose	Treatment	Route	Control measures
Dittgen et al. (2012)	Wistar	Female	Contusion	T9-T10	G-CSF	30 μg/kg	Two weeks after SCI	Hypodermic injection	Excipient
Ha et al. (2005)	SD	Male	Contusion	T9	GM-CSF	20 mg/kg	Five consecutive days after SCI	Hypodermic injection	PBS
Kadota et al. (2012)	SD	Female	Contusion	T9	G-CSF	15 mg/kg	Five consecutive days after SCI	Intravenous injection	Physiological saline
Kawabe et al. (2011)	SD	Female	Contusion	T8-T9	G-CSF	15 mg/kg	Five consecutive days after SCI	Intravenous injection	Physiological saline
Lee et al. (2012)	SD	Female	Contusion	T10	G-CSF	100 μg/kg	Five consecutive days from the ninth day after SCI	Hypodermic injection	Physiological saline
Osada et al. (2010)	C57BL/6	None	Applying a base plate	T8-T9	G-CSF	300 μg/kg	10 consecutive days after SCI	Hypodermic injection	PBS
Park et al. (2020)	SD	Male	Vascular clip	T9	G-CSF	20 μg/kg	Five consecutive days after SCI	Intraperitoneal injection	None
Pitzer et al. (2010)	C57BL/6	Female	Hemisection	T8-T9	G-CSF	1 mg/kg	Two weeks after SCI	Hypodermic injection	Excipient
Teixeira et al. (2018)	Wistar	Male	Contusion	T10	G-CSF	15 mg/kg	Five consecutive days after SCI	Hypodermic injection	None
Urdziková et al. (2011)	Wistar	Male	Balloon compression	T8-T9	G-CSF	50 μg/kg	Five consecutive days after SCI	Intravenous injection	Physiological saline
Chen et al. (2015)	Wistar	Female	Contusion	T10	G-CSF	0.3 mg/kg	24 h after SCI	Intrathecal injection	PBS
Chung et al. (2014)	SD	Male	Vascular clip	T9	G-CSF	70/μg/kg	Injected once immediately after SCI	Intraperitoneal injection	PBS
Guo et al. (2012)	SD	Male	Contusion	T10	G-CSF	20 μg/kg	Seven consecutive days after SCI	Hypodermic injection	PBS
Li et al. (2009)	SD	Half male and half female	Contusion	T10	G-CSF	20 mg/kg	Seven consecutive days after SCI	Hypodermic injection	PBS
Hayashi et al. (2009)	BALB/c	Female	Hemisection	T8	G-CSF	17 μL/kg	Injected once immediately after SCI	Gelfoam	Physiological saline
Huang et al. (2007)	SD	Male	Vascular clip	T7-T8	GM-CSF	65 μg/kg	Injected once immediately after SCI	Intraperitoneal injection	PBS
Kato et al. (2015)	SD	Male	Contusion	T9	G-CSF	15 μg/kg	Five consecutive days after SCI	Intraperitoneal injection	Physiological saline
Huang et al. (2009)	SD	Male	Vascular clip	T9	GM-CSF	70/μg/kg	Injected once immediately after SCI	Intraperitoneal injection	PBS
Guo et al. (2014)	Kunming mice	Female	Hemisection	T10	G-CSF	50 μg/kg	Three consecutive days after SCI	Hypodermic injection	PBS
Guo et al. (2015)	Kunming mice	Female	Hemisection	T10	G-CSF	50 g/kg	Three consecutive days after SCI	Hypodermic injection	PBS

### Risk of bias and quality assessment of the included studies

The risk of bias was assessed, and SYRCLE’s RoB tool was utilized to analyze article quality. A total of 13 studies described blinding of the outcome assessors; one study described blinding of caregivers and/or investigators. No studies reported random sequence generation, allocation concealment, randomization, or binding outcome assessors. Details of the assessment are summarized in Supplementary Figures S1, S2.

### The therapeutic effect of G-CSF on neurological function in spinal cord injury meta-analysis of the effectiveness of G-CSF on neurological function after SCI

**Figure 2:**
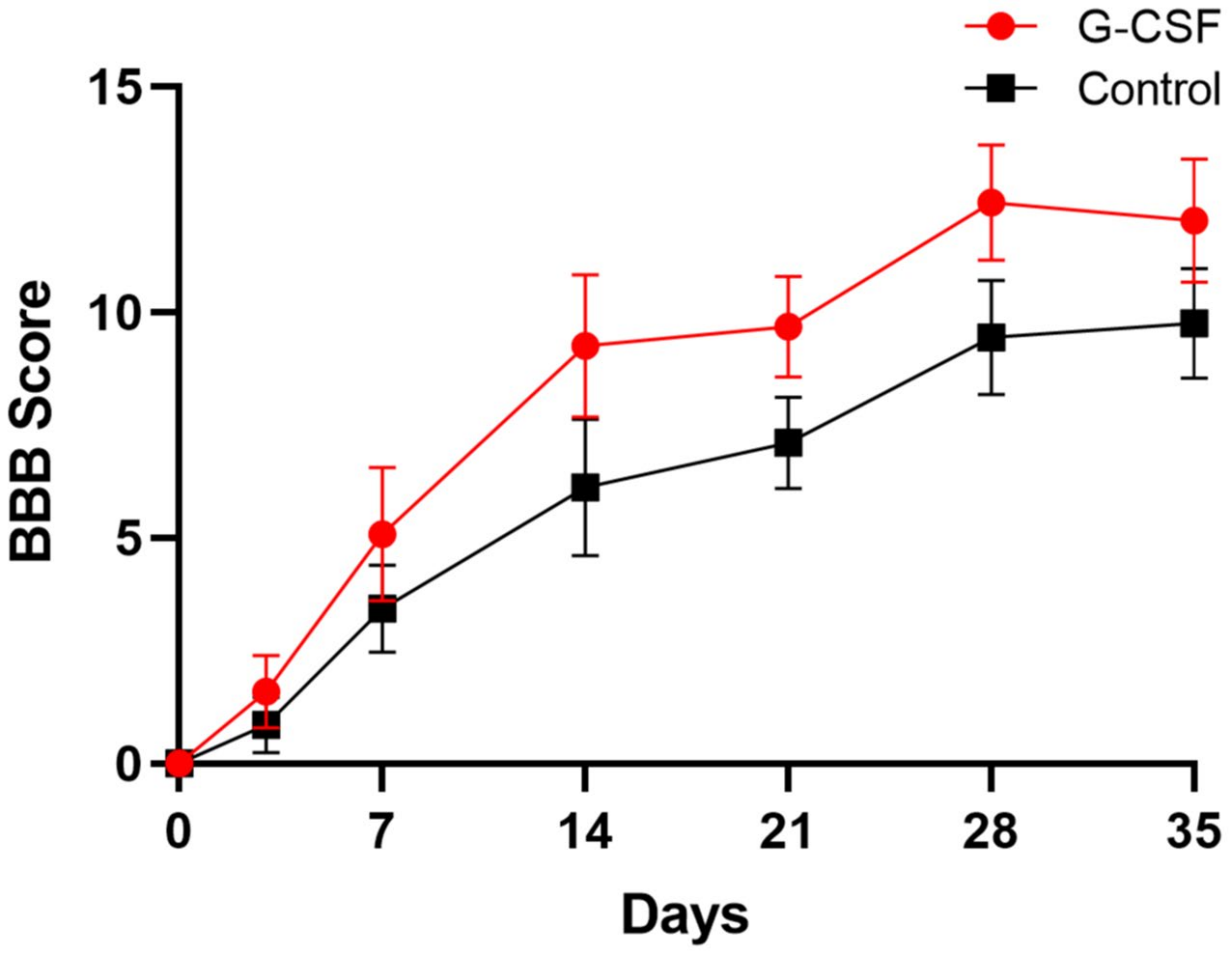
BBB score changes in both groups at 3, 7, 14, 28, and 35  days.

**Figure 3:**
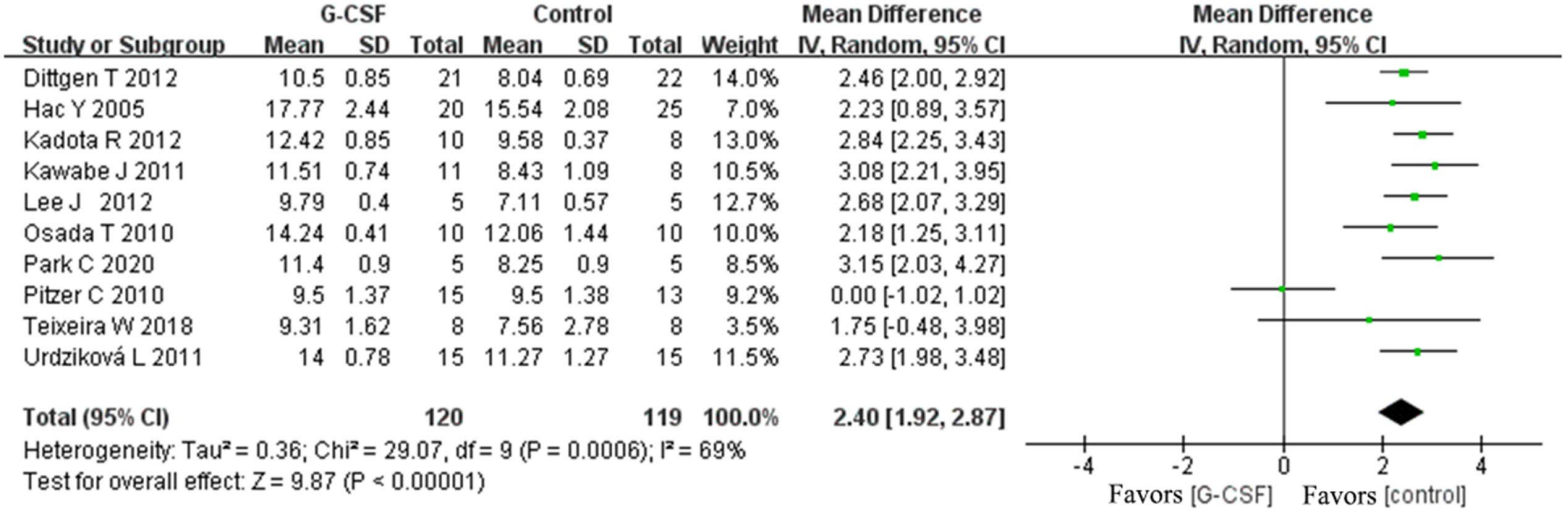
Forest plot of G-CSF effectiveness on the BBB scale scores of animals with SCI at day 35.

**Figure 4:**
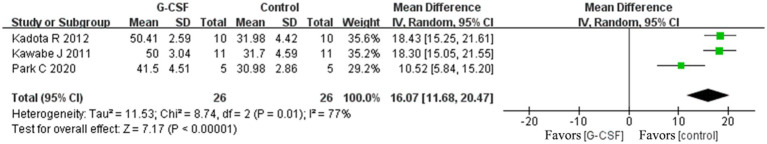
Forest plot of G-CSF effectiveness in inclined plane test scores of animals with SCI.

**Figure 5:**
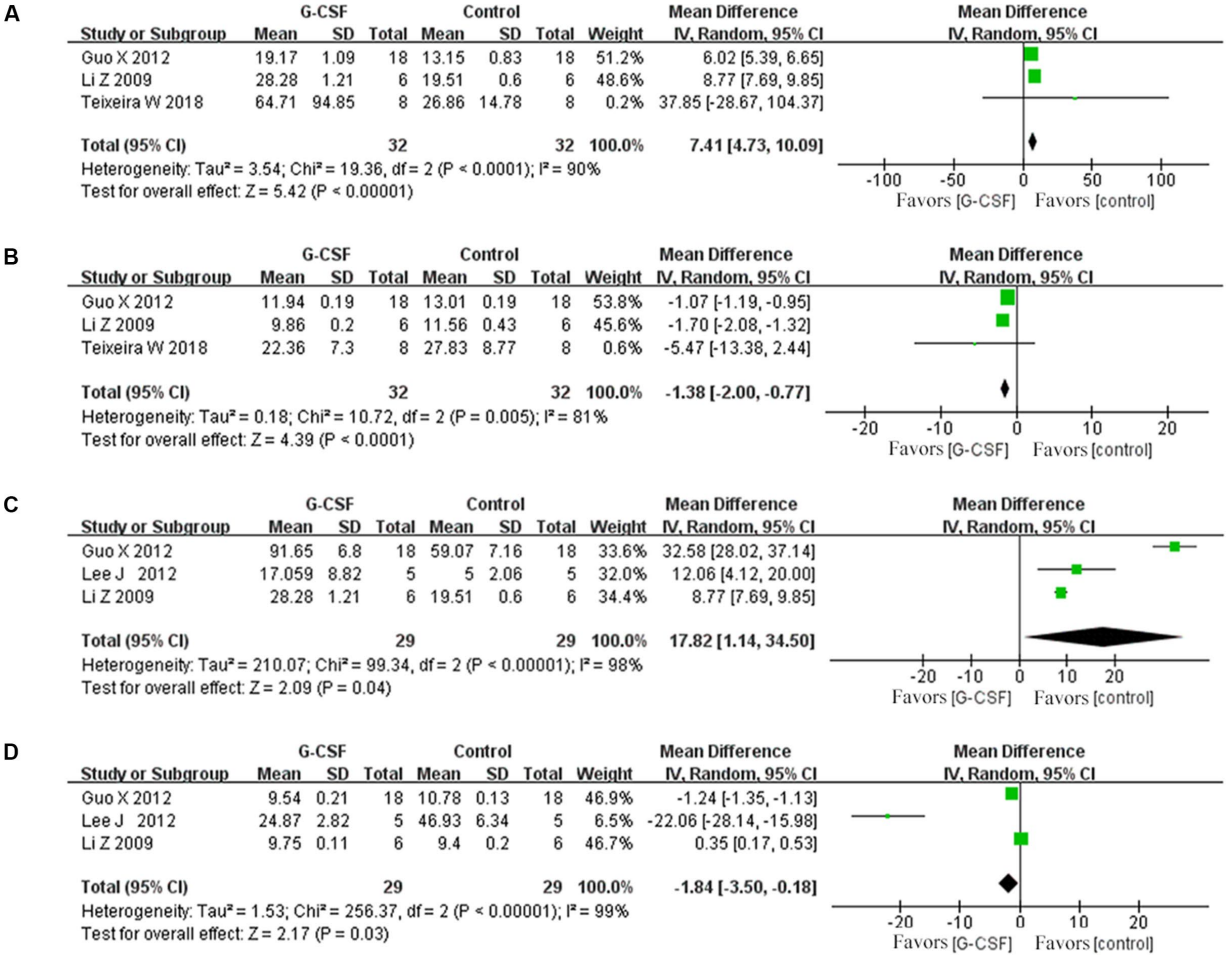
Forest plot of G-CSF effectiveness in the electrophysiological examination of animals with SCI. **(A)** Forest plot of G-CSF effectiveness in motion evoked potential (MEP)-amplitude. **(B)** Forest plot of G-CSF effectiveness in the motion evoked potential (MEP)- latency. **(C)** Forest plot of G-CSF effectiveness in the somatosensory evoked potential (SEP)-amplitude. **(D)** Forest plot of G-CSF effectiveness in the somatosensory evoked potential (SEP)-latency.

**Figure 6:**
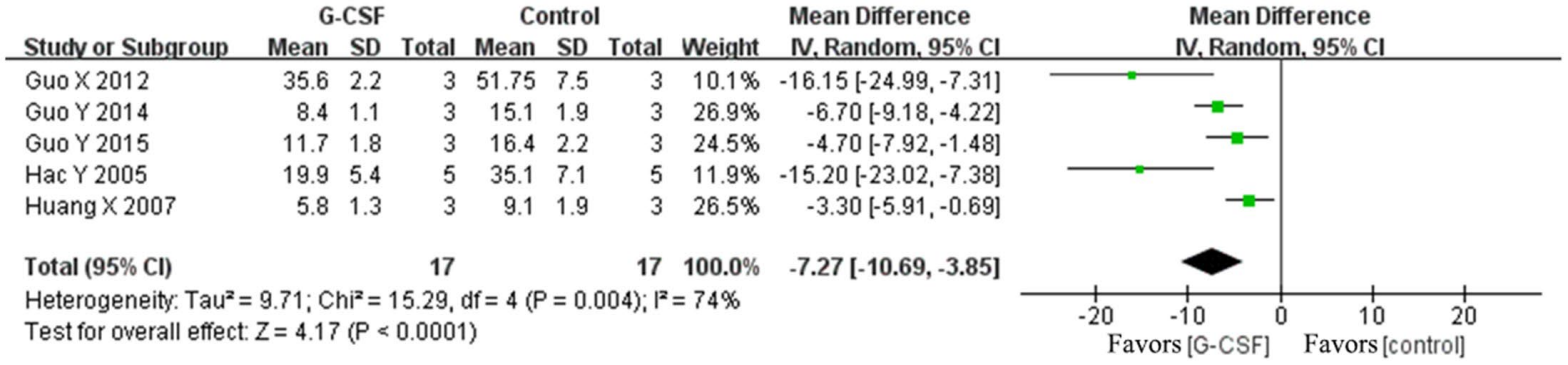
Forest plot of G-CSF effectiveness on TUNEL-positive cell counts in animals with SCI.

**Figure 7:**
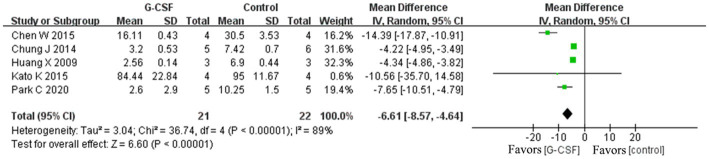
Forest plot of G-CSF effectiveness on GFAP expression in animals with SCI.

According to this meta-analysis, all studies revealed that G-CSF therapy significantly improved motor function in SCI animal models (from day 3 to day 35, Figure 2). By combining the final indicators of 10 articles, it was concluded that the BBB score increased significantly after G-CSF therapy on day 35 (Figure 3). Three articles in the meta-analysis indicated that G-CSF therapy can significantly increase inclined plane scores (Figure 4). Four studies in the meta-analysis indicated that electrophysiologic examination results were significantly improved after G-CSF therapy (Figures 5A–D). Five studies in the meta-analysis indicated that G-CSF therapy considerably reduced the number of TUNEL-positive cells (Figure 6). The conclusion pooled from five articles showed that the quantitative analysis of GFAP immunostaining images was significantly reduced after G-CSF medication (Figure 7).

### Effect of the control group treatment method on BBB scores

A subgroup analysis based on the control group’s treatment strategy at day 35 was performed. Treatment with normal saline and no treatment resulted in significantly improvements in neurological function compared to the G-CSF therapy. The effectiveness of the excipient treatment in the animals was not as pronounced as that of G-CSF (Supplementary Figure S3A). Our subgroup analysis revealed that the heterogeneity might be attributable to the treatment method used for the control group (Table 2).

**Table 2:** Subgroup analysis of the effects of G-CSF on BBB scores.

Subgroup title	No. of studies	Weighted mean difference		Heterogeneity	
		95% Cl	*p*-value	*I* ^2^	*p*-value
1 Species					
1.1 BBB score on day 3	7	0.71 [0.36, 1.06]	0.002	94.8	<0.00001
1.1.1 SD rats	4	1.21 [0.16, 2.26]	0.024	93.4	<0.00001
1.1.2 Wistar rats	2	0.41[−0.45, 1.27]	0.35	79.8	0.026
1.1.3 Kunming mice	1	0.62 [0.33, 0.91]	<0.00001	/	/
1.2 BBB score on day 7	17	1.77 [1.65, 1.89]	<0.00001	97.3	<0.00001
1.2.1 SD rats	9	1.80 [1.64, 1.97]	<0.00001	98.2	<0.00001
1.2.2 Wistar rats	4	1.59 [1.35, 1.83]	<0.00001	97.3	<0.00001
1.2.3 C57BL/6 mice	2	1.40 [0.96, 1.83]	<0.00001	91.6	0.001
1.2.3 BALB/C	1	2.39 [1.91, 2.87]	<0.00001	/	/
1.2.4 Kunming mice	1	1.90 [1.49, 2.31]	<0.00001	/	/
1.3 BBB score on day 14	16	3.14 [2.98, 3.29]	<0.00001	98.8	<0.00001
1.3.1 SD rats	9	3,57 [3.35, 3.80]	<0.00001	98.3	<0.00001
1.3.2 Wistar rats	4	3.58 [3.27, 3.89]	<0.00001	99.6	<0.00001
1.3.3 C57BL/6 mice	2	2.27 [1.74, 2.81]	<0.00001	74.4	0.048
1.3.4 BALB/C	1	1.99 [1.66, 2.32]	<0.00001	/	/
1.4 BBB score on day 21	12	2.36 [2.14, 2.58]	<0.00001	61.1	0.003
1.4.1 SD rats	6	2.60 [2.24, 2.96]	<0.00001	59.9	0.029
1.4.2 Wistar rats	4	2.14 [1.84, 2.43]	<0.00001	70.6	0.017
1.4.3 C57BL/6 mice	2	2.71 [2.01, 3.41]	<0.00001	0	0.377
1.5 BBB score on day 28	16	3.11 [2.95, 3.27]	<0.00001	97.5	<0.00001
1.5.1 SD rats	9	3.21 [3.02, 3.39]	<0.00001	98.0	<0.00001
1.5.2 Wistar rats	4	2.52 [2.06, 2.98]	<0.00001	82.3	0.001
1.5.3 C57BL/6 mice	2	5.14 [4.61, 5.68]	<0.00001	98.4	<0.00001
1.5.4 BALB/C mice	1	0.24 [−0.40, 0.88]	0.462	/	/
1.6 BBB score on day 35	10	2.40 [1.92, 2.87]	<0.00001	69	0.0006
1.6.1 SD rats	5	2.82 [2.47, 3.16]	<0.00001	0	0.80
1.6.2 Wistar rats	3	2.51 [2.12, 2.90]	<0.00001	0	0.66
1.6.3 C57BL/6 mice	2	1.10 [−1.04, 3.24]	0.31	90	0.002
2 Treatment method with the control group					
2.1 BBB score on day 3	7	0.71 [0.36, 1.06]	0.002	94.8	<0.00001
2.1.1 PBS	6	1.32 [0.56, 2.08]	0.788	95.0	<0.00001
2.1.2 None	1	−0.1 [−0.83, 0.63]	0.001	/	/
2.2 BBB score on day 7	17	1.77 [1.65, 1.89]	<0.00001	97.3	<0.00001
2.2.1 Excipient	2	0.84 [0.53, 1.15]	<0.00001	95.9	<0.00001
2.2.2 PBS	8	3.18 [2.98, 3.37]	<0.00001	95.5	<0.00001
2.2.3 Normal saline	5	0.89 [0.72, 1.08]	<0.00001	94.8	<0.00001
2.2.4 None	2	0.95 [0.26, 1.65]	0.007	59.5	0.116
2.3 BBB score on day 14	16	3.14 [2.98, 3.29]	<0.00001	98.8	<0.00001
2.3.1 Excipient	2	2.06 [1.64, 2.49]	<0.00001	81.2	0.021
2.3.2 PBS	7	5.25 [5.01, 5.49]	<0.00001	99.2	<0.00001
2.3.3 Normal saline	5	1.68 [1.45, 1.91]	<0.00001	66.9	0.017
2.3.4 None	2	0.92 [−0.17, 2.01]	0.099	87.9	0.004
2.4 BBB score on day 21	12	2.36 [2.14, 2.58]	<0.00001	61.1	<0.00001
2.4.1 Excipient	2	2.13 [1.77, 2.49]	<0.00001	0	0.466
2.4.2 PBS	4	3.52 [2.90, 4.15]	<0.00001	0	0.566
2.4.3 Normal saline	4	2.30 [1.99, 2.61]	<0.00001	15	0.317
2.4.4 None	2	1.50 [0.22, 2.77]	0.021	81.8	0.019
2.5 BBB score on day 28	16	3.11 [2.95, 3.27]	<0.00001	97.5	<0.00001
2.5.1 Excipient	2	1.59 [1.20, 1.98]	<0.00001	0	0.714
2.5.2 PBS	7	4.69 [4.34, 4.85]	<0.00001	98.3	<0.00001
2.5.3 Normal saline	5	2.31 [2.06, 2.56]	<0.00001	80.5	<0.00001
2.5.4 None	2	2.61 [1.22, 3.99]	<0.00001	66.2	0.085
2.6 BBB score on day 35	10	2.40 [1.92, 2.87]	<0.00001	69	0.0006
2.6.1 excipient	2	1.27 [−1.14, 3.68]	0.3	95	<0.00001
2.6.2 PBS	2	2.20 [1.43, 2.96]	<0.00001	0	0.95
2.6.3 Normal saline	4	2.80 [2.46, 3.14]	<0.00001	0	0.90
2.6.4 None	2	2.80 [1.60, 3.99]	<0.00001	17	0.27
3 SCI segment					
3.1 BBB score on day 3	7	0.71 [0.36, 1.06]	0.002	94.8	<0.00001
3.1.1 T8 or T7-T8	1	2.15 [1.71, 2.60]	<0.00001	/	/
3.1.2 T9 or T8-T9	2	1.36 [0.30, 2.41]	0.012	80.9	<0.00001
3.1.3 T10 or T9-T10	4	0.75 [−0.38, 1.87]	0.192	96.8	0.022
3.2 BBB score on day 7	17	1.77 [1.65, 1.89]	<0.00001	97.3	<0.00001
3.1.1 T8 or T7-T8	2	2.39 [1.98, 2.80]	<0.00001	0	1
3.2.2 T9 or T8-T9	6	1.27 [1.07, 1.48]	<0.00001	95.6	<0.00001
3.2.3 T10 or T9-T10	8	2.04 [1.88, 2.20]	<0.00001	98.3	<0.00001
3.3.4 T11 or T11-T12	1	0.18 [−0.64, 0.99]	0.665	/	/
3.3 BBB score on day 14	16	3.14 [2.98, 3.29]	<0.00001	98.8	<0.00001
3.3.1 T8 or T7-T8	2	1.84 [1.56, 2.13]	<0.00001	64.0	0.095
3.3.2 T9 or T8-T9	6	1.95 [1.64, 2.67]	<0.00001	84.4	<0.00001
3.3.3 T10 or T9-T10	7	4.85 [4.62, 5.10]	<0.00001	99.3	<0.00001
3.3.4 T11 or T11-T12	1	1.89 [1.23, 2.54]	<0.00001	/	/
3.4 BBB score on day 21	12	2.36 [2.14, 2.58]	<0.00001	61.1	0.003
3.4.1 T9 or T8-T9	5	2.53 [2.18, 2.88]	<0.00001	57.8	0.037
3.4.2 T10 or T9-T10	6	2.20 [1.91, 2.48]	<0.00001	68.2	0.014
3,4,3 T11 or T11-T12	1	3.08 [2.14, 2.58]	<0.00001	/	/
3.5 BBB score on day 28	16	3.11 [2.95, 3.27]	<0.00001	97.5	<0.00001
3.5.2 T8 or T7-T8	2	1.59 [1.29, 1.90]	<0.00001	95.5	<0.00001
3.5.2 T9 or T8-T9	6	2.44 [2.08, 2.80]	<0.00001	79.1	<0.00001
3.5.3 T10 or T9-T10	8	4.17 [3.95, 4.39]	<0.00001	98.1	<0.00001
3.6 BBB score on day 35	10	2.40 [1.92, 2.87]	<0.00001	69	0.0006
3.6.1 T9 or T8-T9	5	2.28 [1.18, 3.37]	<0.00001	95	<0.00001
3.7.2 T10 or T9-T10	5	2.52 [2.21, 2.83]	<0.00001	0	0.81
4 Administration dose					
4.1 BBB score on day 3	7	0.71 [0.36, 1.06]	0.002	94.8	<0.00001
4.1.1 <1 mg/kg	3	1.78 [0.96, 2.59]	<0.00001	91.7	<0.00001
4.1.2 >1 mg/kg	4	0.62 [−0.13, 1.36]	0.105	86.9	<0.00001
4.2 BBB score on day 7	17	1.77 [1.65, 1.89]	<0.00001	97.3	<0.00001
4.2.1 <1 mg/kg	10	1.70 [1.54, 1.85]	<0.00001	97.4	<0.00001
4.2.2 >1 mg/kg	7	1.86 [1.68, 2.05]	<0.00001	97.5	<0.00001
4.3 BBB score on day 14	16	3.14 [2.98, 3.29]	<0.00001	98.8	<0.00001
4.3.1 <1 mg/kg	9	2.77 [2.59, 2.95]	<0.00001	98.2	<0.00001
4.3.2 >1 mg/kg	7	4.04 [3.76, 4.33]	<0.00001	99.2	<0.00001
4.4 BBB score on day 21	12	2.36 [2.14, 2.58]	<0.00001	61.1	0.003
4.4.1 <1 mg/kg	6	2.28 [1.99, 2.56]	<0.00001	37.3	0.155
4.4.2 >1 mg/kg	6	2.49 [2.15, 2.83]	<0.00001	74.2	<0.00001
4.5 BBB score on day 28	17	3.11 [2.95, 3.27]	<0.00001	97.5	<0.00001
4.5.1 <1 mg/kg	10	2.86 [2.67, 3.06]	<0.00001	98.1	<0.00001
4.5.2 >1 mg/kg	7	3.64 [3.35, 3.92]	<0.00001	96.3	<0.00001
4.6 BBB score on day 35	10	2.40 [1.92, 2.87]	<0.00001	69	0.0006
4.6.1 <1 mg/kg	5	2.58 [2.28, 2.88]	<0.00001	0	0.69
4.6.2 >1 mg/kg	5	2.03 [0.84, 3.21]	0.0008	85	<0.00001

### Effect of animal species on BBB scores

A subgroup analysis based on the influence of animal species on BBB scores at day 35 was performed. SD rats and Wistar rats with SCI resulted in significant improvements in neurological function. C57BL/6 mice (Supplementary Figure S3B) had no difference in the final effect. Our subgroup analysis revealed that heterogeneity might be attributable to animal species (Table 2).

### Effect of the SCI segment on BBB scores

A subgroup analysis based on the SCI segment on BBB scores at day 35 was performed. The T9–T10 or T10 segment injury model and the T8–T9 or T9 segment injury model (Supplementary Figure S3C) resulted in significant improvements in neurological function. Our subgroup analysis revealed that the heterogeneity might be attributable to the SCI segment (Table 2).

### Effect of dose on BBB scores

A subgroup analysis based on the administration dose and BBB scores at day 35 was performed. The administration dose <1 mg/kg and administration dose ≥1 mg/kg (Supplementary Figure S3D) resulted in significant improvements in neurological function. Our subgroup analysis revealed that heterogeneity might be attributable to the administration dose (Table 2).

### Publication bias

The BBB score data showed no clear funnel plot asymmetry (Supplementary Figure S4). We found no bias in the analysis result (Egger’s test, *p* = 0.42).

### Network meta-analysis of the G-CSF administration dose

**Figure 8:**
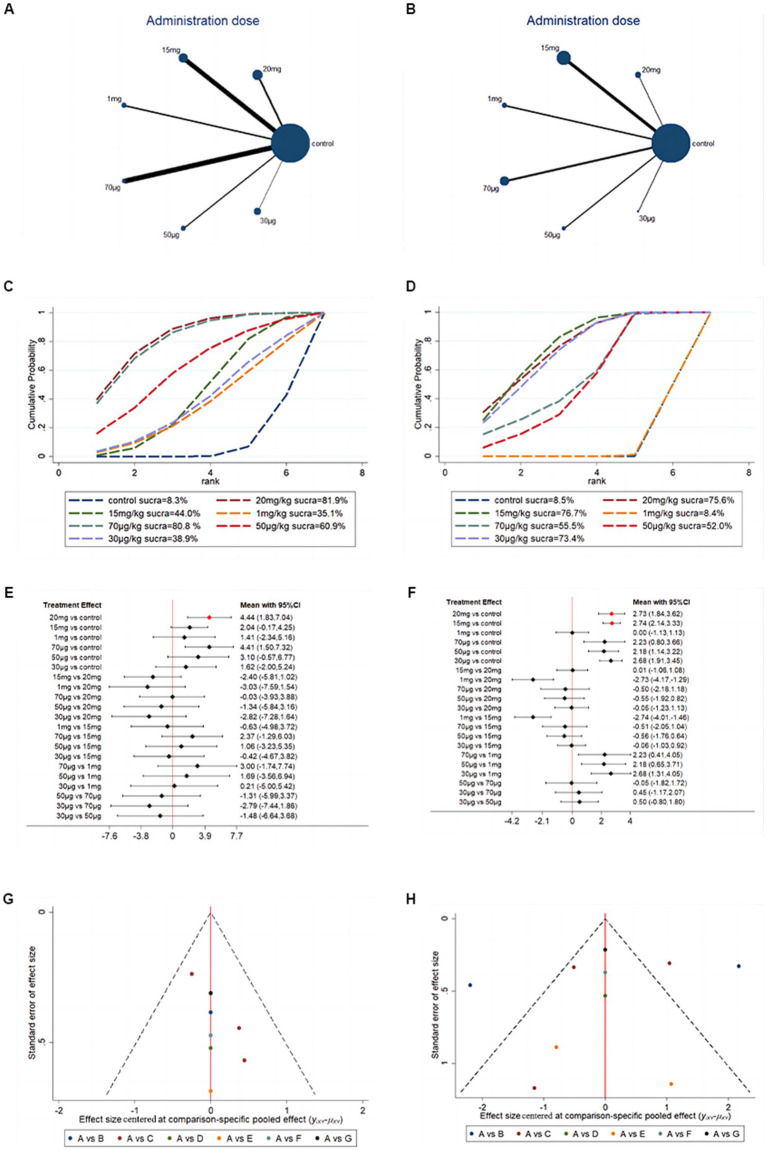
Network meta-analysis based on G-CSF dosage. **(A)** Network evidence map of G-CSF administration dose on day 28. **(B)** Network evidence map of G-CSF administration dose on day 35. **(C)** SUCRA ranking of G-CSF administration dose on day 28. **(D)** SUCRA ranking of G-CSF administration dose on day 35. **(E)** Forest plots of G-CSF administration dose on day 28. **(F)** Forest plots of G-CSF administration dose on day 35. **(G)** Funnel plots of G-CSF administration dose on day 28 [**(A)** 20 mg **(B)** 15 mg **(C)** 1 mg **(D)** 70 μg **(E)** 50 μg  **(F)** 30 μg]. **(H)** Funnel plots of G-CSF administration dose on day 35 [**(A)** 20 mg **(B)** 15 mg **(C)** 1 mg **(D)** 70 μg **(E)** 50 μg  **(F)** 30 μg].

To further investigate the optimal dose of G-CSF for long-term efficacy, network analysis was conducted on the basis of BBB scores at days 28 and 35. The network evidence map suggests that most of the studies in the included studies chose a 15 mg/kg administration dose (Figures 8A,B). Forest plots and pairwise league tables indicated that a 15 mg/kg administration dose on day 35 (MD = 2.74, 95% CI: 2.14 to 3.33, Figure 8C) and a 20 mg/kg administration dose on day 28 (MD = 4.44, 95% CI: 1.83 to 7.04, Figure 8D) were optimal compared with the other doses (Table 3). The SUCRA ranking showed that a 15 mg/kg administration dose on day 35 (SUCRA = 76.7%, Figure 8E) and a 20 mg/kg administration dose on day 28 (SUCRA = 81.9%, Figure 8F) were the optimal doses. G-CSF at high doses may be more effective than at low doses. The funnel plots indicated that publication bias may exist in this network meta-analysis, which may be caused by a small sample size (Figures 8G,H).

**Table 3:** Pairwise league table of different doses administered on days 28 (bottom left) and 35 (upper right).

control	15.33 (6.29,37.37)	15.44 (8.54,27.91)	1.00 (0.32,3.08)	9.30 (2.23,38.70)	8.85 (3.12,25.08)	14.59 (6.73,31.59)
0.01 (0.00,0.16)	**20 mg/kg**	1.01 (0.35,2.93)	0.07 (0.02,0.27)	0.61 (0.11,3.26)	0.58 (0.15,2.27)	0.95 (0.29,3.09)
0.13 (0.01,1.19)	10.98 (0.36,334.43)	**15 mg/kg**	0.06 (0.02,0.23)	0.60 (0.13,2.82)	0.57 (0.17,1.90)	0.94 (0.36,2.50)
0.24 (0.01,10.34)	20.64 (0.22,1978.16)	1.88 (0.02,145.77)	**1 mg/kg**	9.30 (1.51,57.22)	8.85 (1.91,41.03)	14.59 (3.72,57.16)
0.01 (0.00,0.22)	1.03 (0.02,51.03)	0.09 (0.00,3.62)	0.05 (0.00,5.72)	**70 μg/kg**	0.95 (0.16,5.56)	1.57 (0.31,7.94)
0.05 (0.00,1.77)	3.81 (0.04,343.16)	0.35 (0.00,25.21)	0.18 (0.00,34.99)	3.71 (0.03,401.02)	**50 μg/kg**	1.65 (0.45,6.03)
0.20 (0.01,7.41)	16.73 (0.19,1449.26)	1.52 (0.02,106.26)	0.81 (0.00,148.63)	16.28 (0.16,1696.26)	4.39 (0.03,763.16)	**30 μg/kg**

Bold values are different doses of G-CSF administration.

## Discussion

### Summary of evidence

In total, 20 experiments were included in our study. We assessed the BBB score data from day 3 to day 35 because the recovery of motor function was stable at 35 days after SCI. According to the findings of our meta-analysis, G-CSF treatment could significantly improve BBB scales, inclined plane test scores, electrophysiological examination, apoptotic cell counts, and GFAP expression compared with the control treatment. Therefore, the recovery of neurological and motor functions may benefit from G-CSF therapy.

Motor function scores are better in rats than in mice after G-CSF treatment. Different species may exhibit different therapeutic effects. Given that the excipient composition was unclear, their inconsistent composition may have affected the experimental results. Most control groups received normal saline or no treatment. High-segment SCI may result in more severe symptoms than low-segment SCI. The T9–T10 or T10 segment injury model and the T8–T9 or T9 segment injury model resulted in significant neurological function effectiveness. However, in our subgroup analysis, heterogeneity originated from the high-segment SCI model. The majority of studies started treatment within 24 h of SCI. In most studies, animals received treatment daily for 5 days after injury. In several studies, G-CSF was injected once immediately after injury. Only one study started treatment 9 days after SCI. In most studies, hypodermic injection was the main route of drug administration. Several studies used other simple and convenient methods, such as intravenous injections.

In our meta-analysis, the maximum dose of G-CSF used to treat SCI is 20 mg/kg, and the minimum dose of G-CSF used to treat SCI is 20 μg/kg, and each dose resulted in significant changes in the degree of motor function recovery. Each dose has been reported to have long-term therapeutic effects. In our further network meta-analysis investigation, the SUCRA ranking showed that 15 mg/kg-20 mg/kg was the optimal dose of the G-CSF for long-term efficacy. Higher doses of G-CSF may have better efficacy in SCI animal models. However, using pharmaceutical therapies at high doses may have some adverse effects. No studies have been reported on the optimal dose of G-CSF. Our analysis provides a preclinical indication for future researchers to find the best dosage.

### Strengths and limitations

To the best of our knowledge, this work is the first meta-analysis to evaluate the effects of G-CSF in the setting of SCI. Prior to publication, all selected papers underwent extensive peer assessment and were selected from English-language databases. Our meta-analysis demonstrated that G-CSF has a significant effect on neurological function in SCI animal models. This result would be significant for the development of neurorehabilitation drugs with clinical promise. However, several sources of bias must be considered.

Our meta-analysis has several limitations. First, there were no high-quality articles included in our review. All included studies did not report random sequence generation, randomization, blinding of caregivers and/or investigators, allocation concealment, or random outcome assessment. Only six studies described allocation concealment. Only one study described caregiver and/or investigator blinding. Second, we extracted motor function score data from 3 to 35 days from all studies to evaluate the recovery of motor function. BBB scores are predominantly utilized for motor function assessment in rats. However, two studies in our meta-analysis used C57BL/6 mice and BBB scores to evaluate motor function recovery. We used subgroup analysis to explain the possible heterogeneity. Third, our meta-analysis focused on two types of G-CSF. In total, 17 studies treated SCI with G-CSF, and three studies used GM-CSF. The use of different types of colony-stimulating factors may affect the authenticity of the conclusions. Fourth, a small number of studies limits our conclusions. Further studies are required to collect additional high-quality articles to achieve credible results.

### Possible mechanisms of G-CSF inhibition of apoptosis

Spinal cord injury can directly damage spinal cord tissue and alter the spinal cord’s local microenvironment. Secondary injury often leads to neuronal apoptosis, which negatively impacts the neurological and motor functions of the patient. Several researchers have found that SCI can activate apoptosis mediated by caspase-3, SIRT1/AMPK, Wnt/β-catenin, and E2F1/CDK1 pathways (Shi et al., 2021). G-CSF plays a neuroprotective role by promoting the recruitment of neutrophils to the CNS and inhibiting neuronal apoptosis (Guo et al., 2014). Other researchers have found that G-CSF treatment can provide a neuroprotection effect by regulating mTOR and the p70S6K signal pathway (Dumbuya et al., 2020). Furthermore, G-CSF could also downregulate the level of apoptotic-related markers and upregulate anti-apoptotic-related markers, such as BAD, BAX, and Bcl2 (Menzie-Suderam et al., 2018). The same conclusion was reached in this research on the TUNEL-positive cell counts in animals with SCI.

### Possible mechanisms of G-CSF inhibition of glial scar formation

A glial scar is a hypertrophic lesion penumbra formed by reactive astrocytes, microglia, and inflammatory cells (Tran et al., 2022). It inhibits nerve regeneration at the site of the lesion’s parenchyma. GFAP is a biomarker for astrocyte activation. GFAP causes astrocytes to hypertrophy and change their growth density, blocking the regeneration of nerve axons (Zukor et al., 2013). According to several researchers, G-CSF can inhibit GFAP expression while promoting neurotrophic factor production. According to several researchers, G-CSF can inhibit GFAP expression while promoting neurotrophic factor production (Park et al., 2020). Compared with the control group, G-CSF has been described as having better effects on the suppression of glial scars. It creates a favorable environment for nerve regeneration. The long-term therapeutic effect was also found by several researchers on the glial scar. G-CSF could downregulate the GFAP level around the injury site after 30 days (Chen et al., 2015).

### Possible mechanisms and therapeutic prospects of G-CSF In SCI

Recently, the effectiveness of G-CSF in enhancing neurological function has been demonstrated by researchers. It could cross the intact blood–spinal cord barrier. The G-CSF receptor pathway has been revealed to be a neuroprotective and neural tissue repair system in the CNS (Wallner et al., 2015). G-CSF was expressed in many cells, such as astrocytes, neurons, fibroblasts, etc. It is believed to exert broad regulatory effects on numerous cell types (Lu et al., 2014). The neuroprotective effects of G-CSF contribute to the protection of nerve fibers and the activation of the motor system (Khorasanizadeh et al., 2017). G-CSF treatments suppress the formation of GFAP, then inhibit the construction of the physical barrier of glial scars (Park et al., 2020). It can also stimulate stem cell activity, proliferation, and differentiation following transplantation. Several researchers indicated that G-CSF can increase paracrine activity in post-transplant stem cells (Pang et al., 2019). Stem cell transplantation therapy has been a hot topic in recent years. Stem cells have the potential to differentiate into various phenotypes. Stem cell transplantation promotes synapse formation and regeneration of nerve axons. Growth factors and cytokines can influence the activity and differentiation capacity of stem cells through the autocrine loop. Studies have shown that the release of growth factors and cytokines can increase the expression of membrane proteins through the Akt and Erk signaling pathways to enhance the migration ability of stem cells (Cofano et al., 2019). Some studies have suggested that G-CSF suppresses the upregulation of NF-κB related molecules in the CNS. The NF-κB pathway and the activation of autophagy-related molecules, such as ATG3 and ATG7, are closely related. Therefore, G-CSF could induce autophagy and inhibit neuronal apoptosis, thereby providing an effective intervention (Guo et al., 2015).

Some research has been conducted to evaluate the effectiveness of G-CSF as a neuroprotective therapy for neurological and functional improvement in SCI patients. In one RCT study, G-CSF was strongly associated with recovery of the sensory system by subcutaneous administration (Derakhshanrad et al., 2019). Another multicenter RCT study was also conducted. The motor function score of the treatment group significantly increased 1 week after the administration, and this significant increase was maintained until 1 year after the initial observation (Inada et al., 2014). The purpose of the phase III clinical trials was to explore the effectiveness of G-CSF. The results showed that G-CSF therapy improved the ability of neural loops to control the motor system after the intervention (Derakhshanrad et al., 2018).

## Conclusion

The conclusions of our meta-analysis suggest that G-CSF therapy may promote motor function recovery and have a specific neuroprotective effect in SCI animal models. Animal model species, injury segment, dosage, and administration time may affect the research results. However, the molecular mechanism of the therapeutic effect of G-CSF needs further, in-depth research. To establish the therapeutic efficacy of G-SCF, additional high-quality trials are needed.

## Author contributions

J-WT and XF: manuscript drafting, data analysis, and full-text screening. J-YZ: literature retrieval. L-YH, Y-JM, and H-ZB: data extraction. J-PR and YZ: figure preparation. X-HM and LX: manuscript revision. All authors contributed to the article and approved the submitted version.

## Funding

This study was supported by the National Natural Science Foundation of China, with Grant no 81874467, the China Postdoctoral Science Foundation, with Grant no 2022 M710412, “and the Beijing University of Chinese Medicine first-class disciplines construction projects.”

## Conflict of interest

The authors declare that the research was conducted in the absence of any commercial or financial relationships that could be construed as a potential conflict of interest.

## Publisher’s note

All claims expressed in this article are solely those of the authors and do not necessarily represent those of their affiliated organizations, or those of the publisher, the editors and the reviewers. Any product that may be evaluated in this article, or claim that may be made by its manufacturer, is not guaranteed or endorsed by the publisher.

## Supplementary material

The Supplementary material for this article can be found online at: https://www.frontiersin.org/articles/10.3389/fnins.2023.1168764/full#supplementary-material
