# Supplementary figures and images for: Granulocyte colony-stimulating factor effects on neurological and motor function in animals with spinal cord injury: a systematic review and meta-analysis
Source: Front Neurosci. 2023 Jun 28;17:1168764. doi: 10.3389/fnins.2023.1168764 (PMC10338098; doi:10.3389/fnins.2023.1168764)

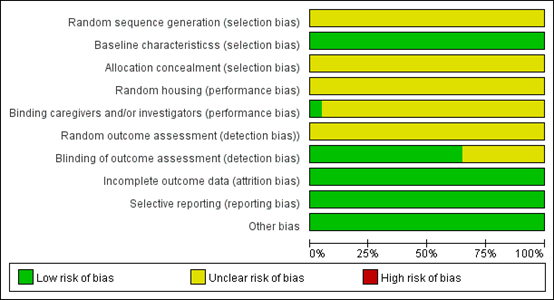

Supplement: Supplementary file 1 [file Image_1.TIF]

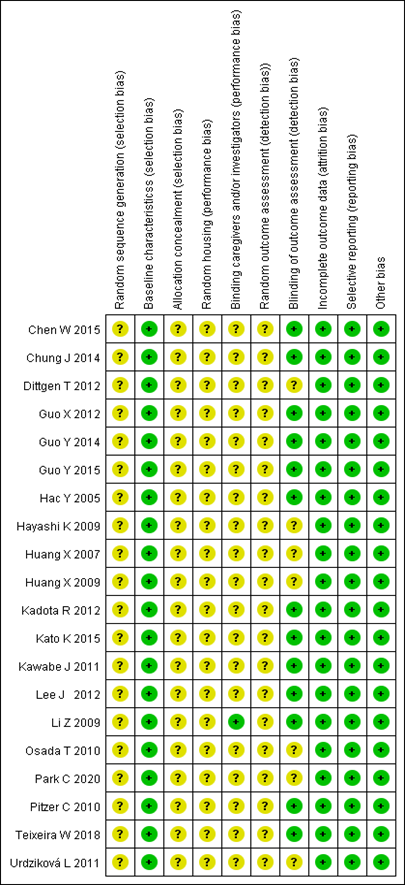

Supplement: Supplementary file 2 [file Image_2.TIF]

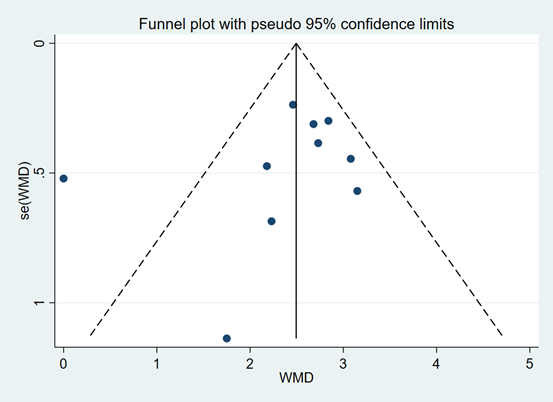

Supplement: Supplementary file 3 [file Image_3.TIF]

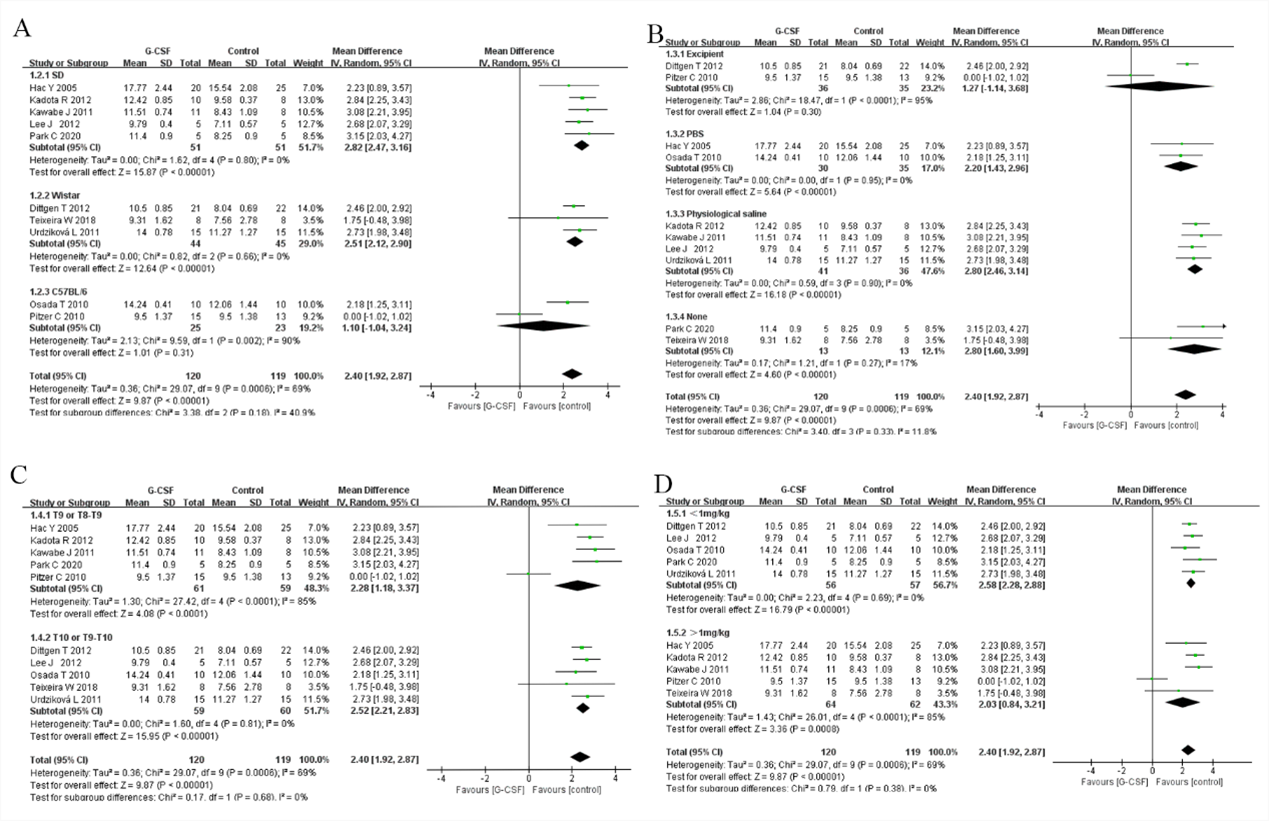

Supplement: Supplementary file 4 [file Image_4.TIF]
